# Fish skin grafts compared to human amnion/chorion membrane allografts: A double‐blind, prospective, randomized clinical trial of acute wound healing

**DOI:** 10.1111/wrr.12761

**Published:** 2019-10-25

**Authors:** Robert S. Kirsner, David J. Margolis, Baldur T. Baldursson, Kristin Petursdottir, Olafur B. Davidsson, Dot Weir, John C. Lantis

**Affiliations:** ^1^ Dr Phillip Frost Department of Dermatology and Cutaneous Surgery University of Miami Miller School of Medicine Miami Florida; ^2^ Department of Dermatology University of Pennsylvania Perelman School of Medicine Philadelphia Pennsylvania; ^3^ Department of Biostatistics and Epidemiology University of Pennsylvania Perelman School of Medicine Philadelphia Pennsylvania; ^4^ Department of Dermatology Landspitali University Hospital of Iceland Reykjavik Iceland; ^5^ Mathematics Division of the Science Institute, University of Iceland Reykjavik Iceland; ^6^ Catholic Health Advanced Wound Healing Centers Buffalo New York; ^7^ Division of Vascular/Endovascular Surgery Mount Sinai St. Luke's—West Hospitals, Icahn School of Medicine New York New York

## Abstract

Chronic, nonhealing wounds consume a great deal of healthcare resources and are a major public health problem, associated with high morbidity and significant economic costs. Skin grafts are commonly used to facilitate wound closure. The grafts can come from the patient's own skin (autograft), a human donor (allograft), or from a different species (xenograft). A fish skin xenograft from cold‐water fish (Atlantic cod, *Gadus morhua*) is a relatively recent option that shows promising preclinical and clinical results in wound healing. Chronic wounds vary greatly in etiology and nature, requiring large cohorts for effective comparison between therapeutic alternatives. In this study, we attempted to imitate the status of a freshly debrided chronic wound by creating acute full‐thickness wounds, 4 mm in diameter, on healthy volunteers to compare two materials frequently used to treat chronic wounds: fish skin and dHACM. The purpose is to give an indication of the efficacy of the two therapeutic alternatives in the treatment of chronic wounds in a simple, standardized, randomized, controlled, double‐blind study. All volunteers were given two identical punch biopsy wounds, one of which was treated with a fish skin graft and the other with dehydrated human amnion/chorion membrane allograft (dHACM). In the study, 170 wounds were treated (85 wounds per group). The primary endpoint was defined as time to heal (full epithelialization) by blinded assessment at days 14, 18, 21, 25, and 28. The superiority hypothesis was that the fish skin grafts would heal the wounds faster than the dHACM. To evaluate the superiority hypothesis, a mixed Cox proportional hazard model was used. Wounds treated with fish skin healed significantly faster (hazard ratio 2.37; 95% confidence interval: (1.75–3.22; *p* = 0.0014) compared with wounds treated with dHACM. The results show that acute biopsy wounds treated with fish skin grafts heal faster than wounds treated with dHACM.

**Abbreviations**
dHACM: dehydrated Human AMNION/Chorion Membrane
FDA: U.S. Food and Drug Administration
PUFA: Polyunsaturated Fatty Acid

## INTRODUCTION

Grafts and materials containing components from processed human and animal tissues are being used in a range of products for the purpose of treating acute wounds and hard‐to‐heal chronic wounds. Fish skin xenografts are a recent addition. The grafts (*Kerecis**®** Omega3 Wound*, Kerecis, Isafjordur, Iceland) from North Atlantic Cod (*Gadus morhua*) are acellular but otherwise structurally intact skin tissue. The grafts are freeze‐dried and possess a shelf life of 3 years. Xenograft materials generally require extensive processing to reduce the risk of viral or prion disease transfer.^1^, ^2^ This processing requires the use of harsh chemicals that dissolve the soluble components of the tissue and denature its structure, leaving behind mostly insoluble collagen.^3^ In the case of cod skin, however, no viral inactivation is necessary.^4^ This allows for milder processing, which retains the structural integrity and the molecular components of the skin, including the proteoglycans, glycoproteins, soluble collagen, elastin, laminin, fibroconectin, lipids, and the Omega‐3 polyunsaturated fatty acids (PUFAs).^5^, ^6^ Previous work has shown these Omega‐3 fatty acids to have bacterial barrier and pain‐modulating properties.^7^, ^8^, ^9^

Amniotic products have gained wide acceptance in wound healing, most specifically in the treatment of diabetic foot ulcers and venous leg ulcers. Of these products, the most commonly used in the United States is dehydrated human amnion/chorion membrane (dHACM; *EpiFix®* MiMedx Group Inc., Marietta, GA, USA). dHACM has been shown to be very effective in treating diabetic foot ulcers.^10^, ^11^, ^12^, ^13^, ^14^ Amniotic membrane‐based allografts are classified in the United States as donated organ components and are currently being used in wound and soft‐tissue repair applications. While those membranes do not undergo viral inactivation, they do undergo vigorous donor screening. Characterization has found growth factors, cytokines, and protease inhibitors within amnio‐chorionic membrane tissues, all of which are suggested to play a role in wound repair.^10^ dHACM is composed of two membranes consisting of multiple layers, including a single layer of nonviable epithelial cells, a basement membrane, and an avascular connective tissue matrix. The membrane undergoes several processing steps, which include cleaning, immersing the material in a solution containing antibiotics with or without rinsing, and drying.^15^

### Primary objective

Chronic wounds vary greatly in etiology and nature, requiring large cohorts for effective comparison between therapeutic alternatives. In this study, we attempted to imitate the status of a freshly debrided chronic wound by creating acute full‐thickness wounds, 4 mm in diameter, on healthy volunteers to compare two materials frequently used to treat chronic wounds. The purpose was to give an indication of the efficacy of the two therapeutic alternatives in the treatment of chronic wounds in a simple, standardized, randomized, controlled, double‐blind study.

The primary objective of the study was a head‐to‐head comparison of the time to heal for full‐thickness wounds on human volunteers using a 4 mm punch biopsy model.

The superiority hypothesis was that the fish skin grafts would be faster in time to heal vs. dHACM. The null hypothesis was therefore that there would have been no significant difference between the treatments.

### Secondary objectives

The secondary objectives were wound healing at day 28, pain, erythema, infection, and the cost of products used.

### Primary endpoints

The primary endpoint was defined as time to heal by blinded assessment of full epithelialization at days 14, 18, 21, 25, and 28.

### Secondary endpoints

The secondary endpoints were the proportion of wounds healed at day 28 as assessed by full epithelialization, incidences of pain, erythema, infection, and a cost comparison derived from the total number of applications of each product.

## MATERIALS AND METHODS

### Study design and participants

The study was a prospective, double‐blinded, randomized, comparative, clinical trial. The research protocol was approved by the Icelandic Medicines Agency (8.1.13.1/2017090010), the Icelandic Data Protection Authority, and the Icelandic National Bioethics Committee (VSNb2018010033/03.01). All participants signed an informed consent form consistent with the World Medical Association (WMA) Declaration of Helsinki statement of ethical principles for medical research involving human subjects.

Recruitment took place over a 2‐month period, and participants were recruited using advertisements at the University of Iceland and the Iceland University of the Arts in Reykjavik, Iceland. Healthy volunteers between the ages of 18 and 60 years were included in the study. Information on demographics, vital signs, and medical history was recorded. Volunteers were excluded prior to randomization if they were using immunosuppressive treatment, anticoagulation therapy (i.e., warfarin), or systemic corticosteroids and if they were suffering from immune deficiency because of disease or iatrogenic, peripheral vascular disease. Women who were pregnant, breast feeding, or planning a pregnancy during the course of the clinical trial were also excluded.

**Figure 1:**
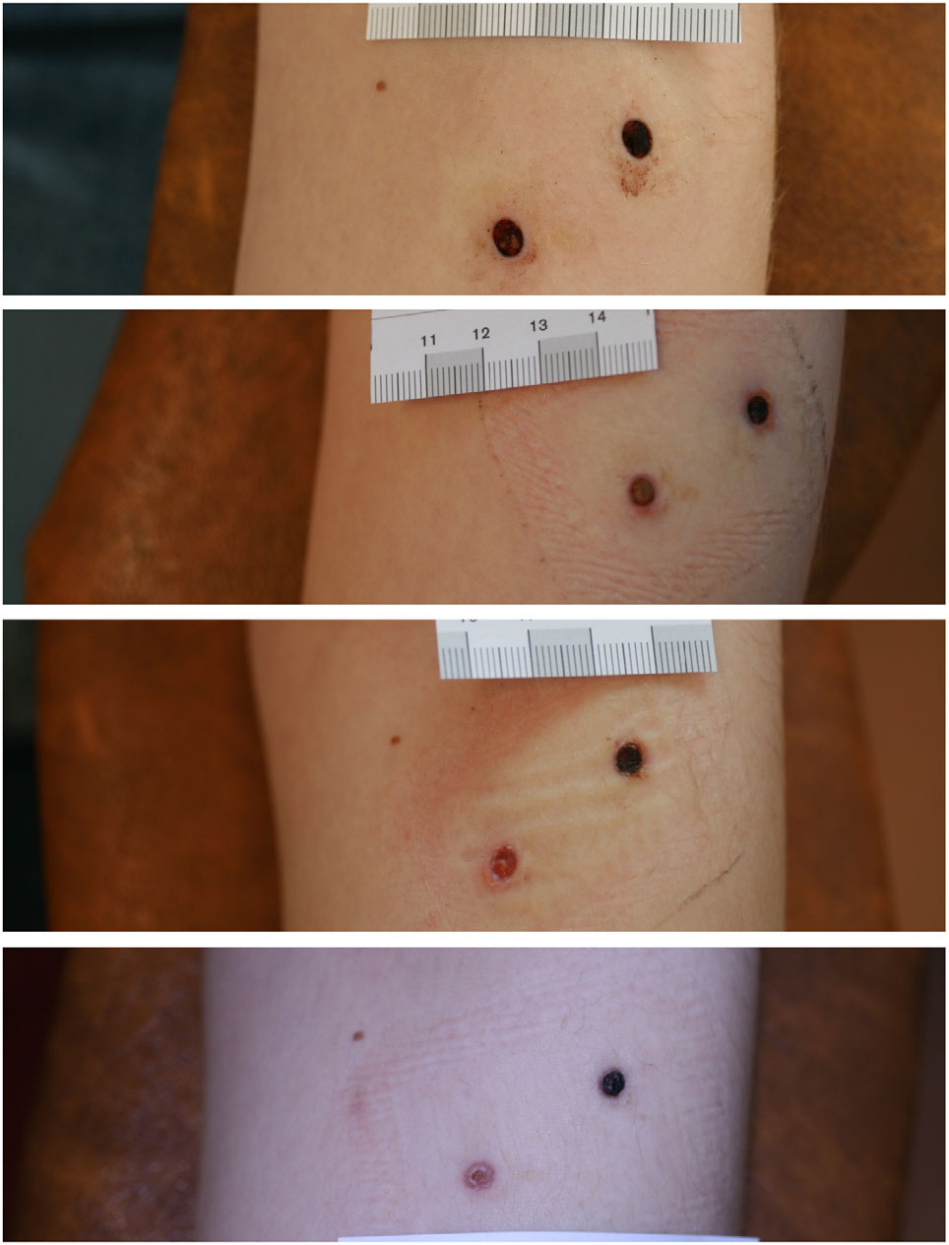
Representative subject followed up to the healing of punch wounds. From top to bottom: D0, D7, D14, D25. Wound on the left side of figure was treated with fish skin graft, while the right wound was treated with dHACM.

Each patient received two full‐thickness, 4 mm punch biopsy wounds created with a standard punch biopsy tool (Figure 1). Participants acted as their own control, with one forearm wound randomized to treatment with fish skin and a second wound on the same forearm treated with dHACM. Two products—fish‐skin graft (*Kerecis® Omega3 Wound*, Kerecis, Isafjordur, Iceland) and dHACM (*EpiFix®* MiMedx Group Inc., Marietta, GA, USA)—were used according to manufacturer's instructions for use.

Two physicians conducted the study; a trial physician applied the products, and an evaluating physician, blinded to the treatment, assessed the wounds and did nothing else. The blinded physician made the clinical assessment of wound closure that, following FDA guidelines, needed to be adjudicated photographically. Three blinded external reviewers then examined all of the photographs of the wounds independently and determined the visit at which closure occurred. Thus four blinded wound‐healing professionals evaluated the wounds.

### Randomization and blinding

All participants in the study were assigned a unique identification number, and all received both treatments. Randomization dictated which wound, laterally or medially located, was treated with which treatment (fish skin or dHACM). The two products were randomly applied using an even/odd randomization scheme. The randomization sequence was accessible to the trial physician and the statisticians of the trial. The patient and the evaluating physician were blinded to the randomization.

### Surgery and trial schedule

After local anesthesia (lidocaine hydrochloride10mg/ml and epinephrine 5 mcg/ml), two full‐thickness, 4 mm punch wounds, 3 cm apart, were made on the proximal anterolateral aspect of the nondominant forearm. Hemostasis was achieved with pressure and a 30% ferrous chloride solution. The products were applied according to the randomization sequence followed by a secondary dressing consisting of a waterproof, vapor‐transmitting, transparent plastic film with a centrally placed gauze pad. Participants were provided with instructions on wound care and two spare secondary dressings to use if necessary before follow up. Participants were instructed not to remove the trial products.

Over the 28‐day study period, including the first study day, participants had a total of seven visits. On day 3, participants were contacted by phone by the trial physician to collect information on the status of their wounds. The participants were seen on days 7, 14, 18, 21, 25, and 28 for assessment of wound status, allowing a visit 1 day later or earlier if necessary.

Wounds were assessed for erythema, infection, and reported pain or bleeding, and a decision of healed vs. not healed was made by the evaluating physician. An a priori erythema up to 1 mm on the wound edge was considered normal. Wider erythema was noted as such, and wider erythema with infiltration with or without exudate that exceeded that of other participants was classified as an infection.

Upon wound inspections, no attempts were made to remove material from the wounds. Wounds with material still visible in the wound were deemed *not healed*. If the material had been resorbed, or if it was not visible, new material was applied to the wound. As the studied materials had no claims of absorption, no attempt was made to evaluate the amount of exudate. In addition to the healed vs. nonhealed decision of the evaluating physician, standardized digital photographs were taken and used for healing assessment (Figure 1). If one of the two wounds was determined to be healed, then no further evaluations were performed on that participant.

### Statistical analysis

The evaluations of four wound‐healing professionals produced a table of outcomes of healed vs. nonhealed wounds for each time point. To estimate whether the differences in time to heal between the studied materials in this accumulated data was significant, a mixed‐effects Cox proportional hazard model was used. The model incorporates a fixed effect for the treatment, a random individual effect to account for each individual receiving both wounds, and a random wound effect to account for the variance introduced by the different reviewers of each wound. All analysis was performed in R version 3.4.1.^16^ The confidence interval for the proportion of wounds healed with each of the treatments at each time point was estimated with the bootstrap method using 2 million simulations. The model parameters were estimated using the “coxme” package in R.^17^ For the cost analysis, a two‐sided paired t‐test was used. A Chi‐square test was used to check for difference in healing in lateral vs. medial wounds.

## RESULTS

### Demography

Eighty‐five (85) healthy volunteers aged between 19 and 51 years were enrolled with no loss of follow up. Their average age was 24.1 years, with a standard deviation of 4.6 years. Two thirds were women. The cohort was primarily of Caucasian origin, consisting of 84 Caucasians and 1 participant of African origin (Table 1).

**Table 1:** Demographic data

Demographic data of study population	
*n = 85*	
Female/male ratio	2.1
Age, average	24.1
Age, range	19–51
Caucasian	98.8%
African	1.2%

### Primary endpoint

**Figure 2:**
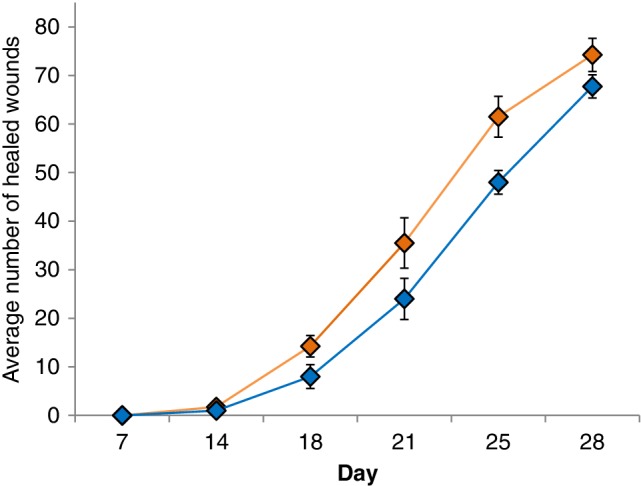
Proportion of healed wounds at each time point with fish skin graft (orange) and dHACM (blue). Wounds treated with fish skin healed significantly faster with a hazard ratio of 2.37 compared to dHACM allograft‐treated wounds (*p* = 0.0014). Projected healing for 50% of wounds was 22 days for fish skin product and 24 days for amniotic product.

**Figure 3:**
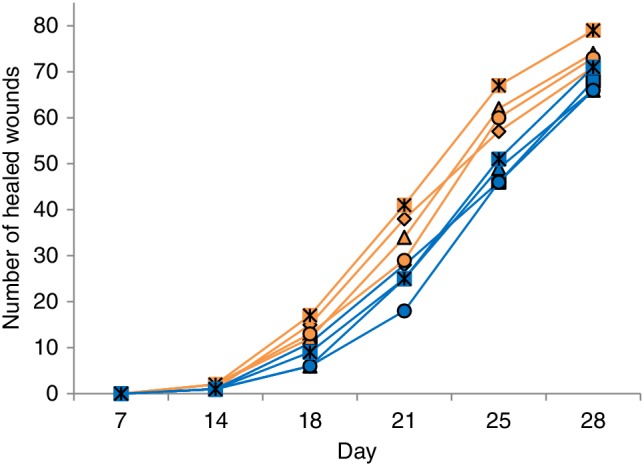
Trajectories of the number of healed wounds to time by evaluator and wound‐healing material. Orange: Intact fish skin. Blue: dHACM.

From day 14 onward, wounds in the group treated with fish skin healed to a greater extent on average than those treated with dHACM (Figure 2). Wounds treated with fish skin healed significantly faster with a hazard ratio of 2.37 (95% CI: 1.75–3.21) and a *p*‐value of 0.0014 compared to dHACM‐treated wounds. Using a likelihood ratio test, other available covariates, such as age and gender, were omitted successively from the model as they did not contribute to a significantly better model fit. Figure 2 shows a greater proportion of healed wounds treated with fish skin, where all reviewers were pooled. Similarly, the healing trajectories of the source materials at each day and per evaluator show consistent superiority of the fish skin grafts (Figure 3). The results of the mixed effects Cox proportional hazard model confirm this difference. The median day of healing in both treatment arms was 25 days.

### Other endpoints

A number of participants reported pruritus (n = 19), and six participants developed a minor rash consistent with the application area of the cover dressing surrounding both wounds and were treated with another brand of cover dressing. Seven patients reported mild pain or discomfort around the wound area, most frequently experienced on the first 2 days. Two participants still felt mild pain on day 3, and one reported intermittent pain up to day 14 without infection or other identifiable causes or connections with other symptoms. Periwound erythema >1 mm was noted in 10 wounds; four subjects had erythema in both wounds, and two had erythema only in the wound treated with fish skin. Thus, differences were not attributable to either treatment type (Table 2). Two subjects (three wounds) experienced irritation with discharge, one in the dHACM wound and the other in both wounds. Two subjects exhibited hypergranulation in one wound, one from each treatment arm. There was no case of infection that needed treatment with antibiotics; the symptoms had subsided at the next dressing change. The fish skin graft received 1.6 applications per subject on average, while the dHACM received 1.4 applications per wound.

**Table 2:** Incidences of erythema, irritation, and infection at a given time point (Day) over the course of the trial

	dHACM, number of wounds (day of trial)	Fish skin, number of wounds (day of trial)
Erythema	2 (7), 2 (14)	4 (7), 2 (14)
Irritation and discharge	1 (7), 1 (14)	1 (7)
Infection	0	0

Of the 47 subjects where one wound healed before the other, by the indication of one or more evaluators, 20 were on the lateral side and 27 on the medial side. The difference between them was not significant (*p* = 0.23).

## DISCUSSION

Full‐thickness acute wounds treated with fish skin grafts heal significantly faster than wounds treated with dehydrated human amnio‐chorionic membrane. There was no difference in the number of adverse reactions, such as mild erythema or irritation, between groups.^18^, ^19^ A previously published clinical trial of similar design showed that full‐thickness acute wounds treated with fish skin heal significantly faster than wounds treated with porcine tissue.^4^ The two studies show that fish skin grafts enable faster healing of acute wounds than amniotic tissue and porcine tissue.

The wound‐healing scores vary between evaluators but show a consistent difference between treatments (Figure 3). Several statistical methods exist that are frequently used to analyze time‐to‐event curves. One of them is the Cox proportional hazard model, which has been widely used in medical research.^20^ This methodology allows data from participants with only one wound healed to be used.

While the exact role of fish skin in accelerated healing of these wounds requires further study, specific properties of the fish skin are likely to be important to this result. Unlike the manufacturing processes for mammalian tissues, the gentle processing of the cod skin does not include viral deactivation, preserving the natural structural and the molecular components of the cod skin. The preservation of the natural three‐dimensional extracellular matrix structure and porosity may be important for cellular recognition. dHCAM relies on the presence of fetal‐derived growth factors.^10^ While there are reports of dHCAM healing chronic diabetic foot ulcers in 1 week, this rapid healing effect was not noted in our acute wound study.^14^ The total fatty acid content of cod skin consists of >30% Omega‐3 PUFAs, while human skin, amnion membrane, and collagen matrix contains 0 or < 1% Omega‐3 of total fatty acid content.^21^ Both of these products have similar application profiles in that they often require five or more applications to close a chronic wound.^8^ Therefore, if one has a significantly higher or lower cost than the other, this difference will increase rapidly with a multiple application strategy.

In this model, the differences in the cost of the product also made the cost of treating wounds with dHACM significantly higher than treating with fish skin (*p* = 2.51*10^−16^). dHACM‐treated wounds were, on average, 76% more expensive compared to fish skin‐treated wounds in this study. The cost was calculated using the average sales price of dHACM ($160 USD/square cm based on 2018 Healthcare Common Procedure Coding System code Q4131) and the average listed sales price of fish skin ($80 USD/square cm).

This study was designed to minimize the effect of individual variance between wounds by studying uniform, acute wounds in healthy patients. When chronic wounds are treated with products such as the fish skin graft and dHACM, they need to be thoroughly debrided. With debridement, the chronic wounds approach the physiological state of an acute wound.^22^ Therefore, results derived from studying acute wounds can have logical implications for debrided chronic wounds.

## LIMITATIONS

When compared head to head to commonly used products, fish skin has been shown to improve time to closure. However, the routine need for reapplication for these products in clinical settings and the nature of the chronic wound patient cannot be reflected in this study design. Overall, the product has shown superiority in this particular model; however, clinical trials on chronic wounds are required to further validate these results.
